# Biocompatibility and Biological Performance of Additive-Manufactured Bioabsorbable Iron-Based Porous Interference Screws in a Rabbit Model: A 1-Year Observational Study

**DOI:** 10.3390/ijms232314626

**Published:** 2022-11-23

**Authors:** Chien-Cheng Tai, Yu-Min Huang, Chen-Kun Liaw, Kuo-Yi Yang, Chun-Hsien Ma, Shin-I Huang, Chih-Chieh Huang, Pei-I Tsai, Hsin-Hsin Shen, Jui-Sheng Sun, Chih-Yu Chen

**Affiliations:** 1 International Ph.D. Program for Cell Therapy and Regeneration Medicine, College of Medicine, Taipei Medical University, Taipei 11031, Taiwan; 2 Department of Orthopedics, Shuang Ho Hospital, Taipei Medical University, New Taipei City 23561, Taiwan; 3 Department of Orthopedics, School of Medicine, College of Medicine, Taipei Medical University, Taipei 11031, Taiwan; 4 Graduate Institute of Biomedical Optomechantronics, College of Biomedical Engineering, Research Center of Biomedical Device, Taipei Medical University, Taipei 11031, Taiwan; 5 Biomedical Technology and Device Research Laboratories, Industrial Technology Research Institute, Chutung, Hsinchu 31057, Taiwan; 6 Department of Orthopedics, China Medical University, Taichung 40202, Taiwan; 7 School of Medicine, College of Medicine, China Medical University, Taichung 40202, Taiwan; 8 International Ph.D. Program in Biomedical Engineering, College of Biomedical Engineering, Taipei Medical University, Taipei 11031, Taiwan

**Keywords:** additive manufacturing (3D printing), bioabsorbable, iron-based, interference screw

## Abstract

This study evaluated the mid-term (12-month) biomechanical, biocompatibility, and biological performance of additive-manufactured bioabsorbable iron-based interference screws (ISs). Two bioabsorbable iron IS types—manufactured using pure iron powder (iron_IS) and using pure iron powder with 0.2 wt% tricalcium phosphate (TCP_IS)—were compared with conventional metallic IS (control) using in vitro biocompatibility and degradation analyses and an in vivo animal study. The in vitro ultimate failure strength was significantly higher for iron_IS and TCP_IS than for control ISs at 3 months post-operatively; however, the difference between groups were nonsignificant thereafter. Moreover, at 3 months after implantation, iron_IS and TCP_IS increased bone volume fraction, bone surface area fraction, and percent intersection surface; the changes thereafter were nonsignificant. Iron_IS and TCP_IS demonstrated degradation over time with increased implant surface, decreased implant volume, and structure thickness; nevertheless, the analyses of visceral organs and biochemistry demonstrated normal results, except for time-dependent iron deposition in the spleen. Therefore, compared with conventional ISs, bioabsorbable iron-based ISs exhibit higher initial mechanical strength. Although iron-based ISs demonstrate high biocompatibility 12 months after implantation, their corrosive iron products may accumulate in the spleen. Because they demonstrate mechanical superiority along with considerable absorption capability after implantation, iron-based ISs may have potential applications in implantable medical-device development in the future.

## 1. Introduction

Inert metallic biomaterials have long been used in medical applications owing to their satisfactory mechanical properties, excellent corrosion resistance, and ease of production [1,2]. These devices are designed for load-bearing and permanent implantation within the body, unless they require interventional removal [3,4]. However, when used for mechanical support, permanent inert materials can cause several complications over time; these may include hypersensitivity reactions, foreign body sensation, implanted tissue weakening due to stress shielding over time, diagnostic image interferences, and even secondary surgery for implant removal [2,3,4,5]. Therefore, a perfect strategy to avoid these complications may include the use of clinically safe and effective, self-degradable metallic implants; they can be used for initial support and then for gradually transferring load toward the healing tissues until complete recovery.

Most commercially available clinical bioabsorbable implants composed of biodegradable synthetic polymer compounds have been approved by regulatory authorities worldwide [4,6]. However, typical biodegradable synthetic polymer-based implants may not have sufficient stiffness suitable for major load-bearing scenarios [7,8]. Recent technological advances have enabled the development of biodegradable metallic material-based implants with significantly higher mechanical strength and the potential to revolutionize metallic implant design and treatment strategies [4,5,9,10,11].

The corrosion and biocompatibility of three classes of current biodegradable metals, namely magnesium (Mg), zinc (Zn), and iron (Fe), have been investigated extensively [12,13,14]. Of these, Fe-based biodegradable materials are considered suitable candidates for use as metallic implants because they have been demonstrated to have satisfactory cytocompatibility and mechanical properties similar to those of natural bone [14,15,16,17,18]. However, the major limitation impeding the clinical application of Fe-based biodegradable materials are their tendency to slowly degrade in physiological environments [5,15,16,19]. To facilitate rapid degradation of iron-based biodegradable materials, several potential strategies have been reported; one of them involves incorporating a porous structure design into the implants [5,20,21,22], whereas another requires the use of a composite material rather than pure iron [23,24,25]. According to our previous work, a porous structure was integrated in the screw design in order to increase roughness and porosity rate [20]. Ulum et al. demonstrated that iron-based bioceramic (hydroxyapatite, tricalcium phosphate (TCP), and biphasic calcium phosphate) had a better degradation rate [23]. Thus far, most studies on the clinical biocompatibility of iron-based bioabsorbable implants have been in vivo [25]. Studies have reported that iron from implants is typically metabolized safely within a host body [3,12,13,14,25]. Similarly, we previously reported no detectable adverse metabolic responses in our animal study on short-term (3-month) biocompatibility of iron-based bioabsorbable implants [26]. However, longer-term clinical biocompatibility animal studies to understand the long-term effects of iron and its corrosion products in the body are warranted.

In the current study, we developed two types of bioabsorbable iron-based porous interference screws (ISs) utilizing additive manufacturing (AM) by using (1) pure iron powder (iron_IS) and (2) pure iron powder plus TCP (TCP_IS). We then evaluated their mid-term (12-month) biocompatibility; biomechanical performance; and results of micro-computed tomography (CT) results, histopathological analysis at implanted sites, serial host serum biochemical analysis, and histopathological analyses of essential organs (heart, liver, spleen, lungs, and kidneys at sacrifice) in a rabbit model. To our knowledge, this is the first in vivo animal report to evaluate the mid-term metabolic route and degradation profile of bioabsorbable iron-based porous implants.

## 2. Results

### 2.1. In Vitro Biocompatibility Analyses of Bioabsorbable ISs

**Figure 1:**
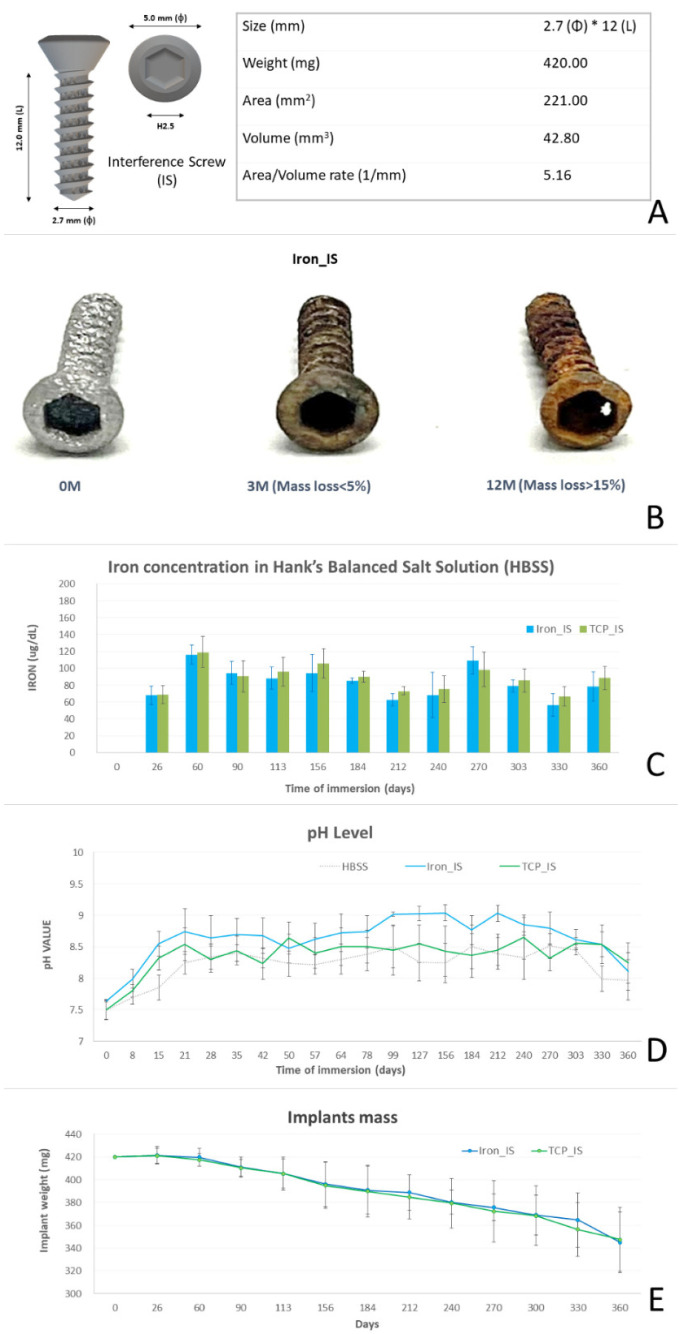
(**A**) Specifications of interference screws (ISs) used in this study. (**B**) Gross appearance of a pure iron bioabsorbable interference screw from an in vitro immersion test in a 12-month period. (**C**–**E**) Fe concentration, pH, and implants mass loss of iron_IS and TCP_IS from an immersion test in Hank’s balanced salt solution over 12 months. Error bars represent standard deviations.

The specifications and gross appearances of the ISs are illustrated in Figure 1A. The gross appearance of iron_IS after the in vitro immersion test over the 12-month period is presented in Figure 1B. Iron concentration (µg/dL), pH level, and mass loss of iron_IS and TCP_IS immersed in HBSS over 12 months are shown in Figure 1C–E and Table 1.

**Figure 2:**
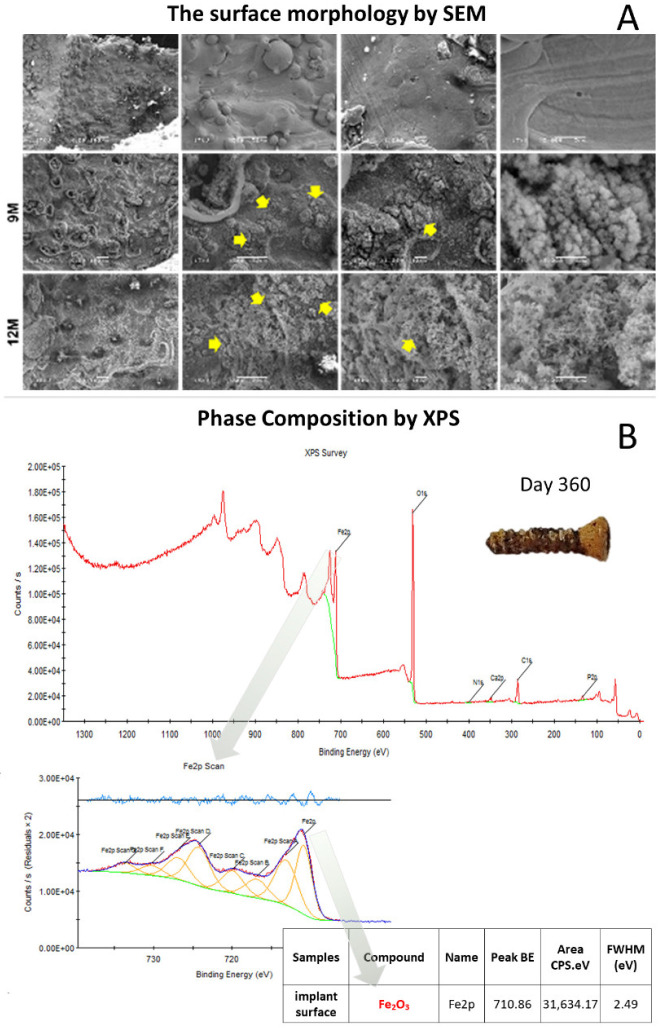
Surface morphology of Fe-based bioabsorbable interference screws retrieved on day 360 after in vitro immersion test observed using (**A**) scanning electron microscopy (SEM) and (**B**) phase composition X-ray photoelectron spectroscopy (XPS). The yellow arrows in (**A**) indicate surface-degraded iron oxides in the forms of needles and spheres. Fe_2_O_3_ is the major compound identified using phase composition XPS in (**B**).

Next, we assessed the surface morphology of iron_IS after the immersion test over 12 months using SEM and observed surface-degraded iron oxides in the form of needles and spheres (Figure 2A). In addition, the phase composition analysis using XPS demonstrated Fe_2_O_3_ to be the main iron oxide (Figure 2B).

### 2.2. In Vivo Biomechanical Analysis

**Figure 3:**
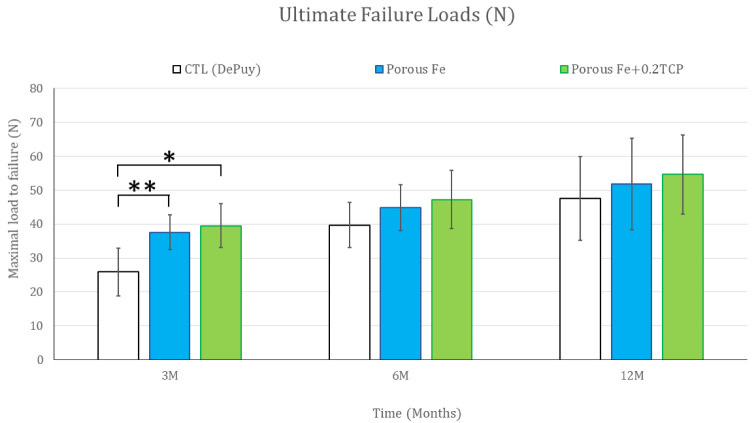
Ultimate failure loads of the bone–tendon–screw complex in vivo. Ultimate failure loads in all study groups increased incrementally throughout the study period. They were significantly higher in the iron_IS and TCP_IS groups than in the control group 3 months after implantation (*p* = 0.0025 and 0.026, respectively). Error bars represent standard deviations. * and ** denote significant differences between groups.

The ultimate failure loads of the bone–tendon–screw complex (Figure 3 and Table 2) were higher in the iron_IS and TCP_IS groups than in the control group at post-implantation month 3, but not at post-implantation months 6 and 12. The ultimate failure loads did not differ significantly between the iron_IS and TCP_IS groups throughout the study period. Nevertheless, the ultimate failure loads of all study groups increased incrementally throughout the study period. All specimens failed at the bone–tendon–screw junction area. The ultimate failure loads were significantly higher in the iron_IS and TCP_IS groups than in the control group at post-implantation month 3 (*p* = 0.026). The progressive increase in IS strength was slightly correlated with the degree of bone ingrowth and mineralization and healing tissue maturation, as noted in the histological and micro-CT study subsequently

### 2.3. Micro-CT Analysis

**Figure 4:**
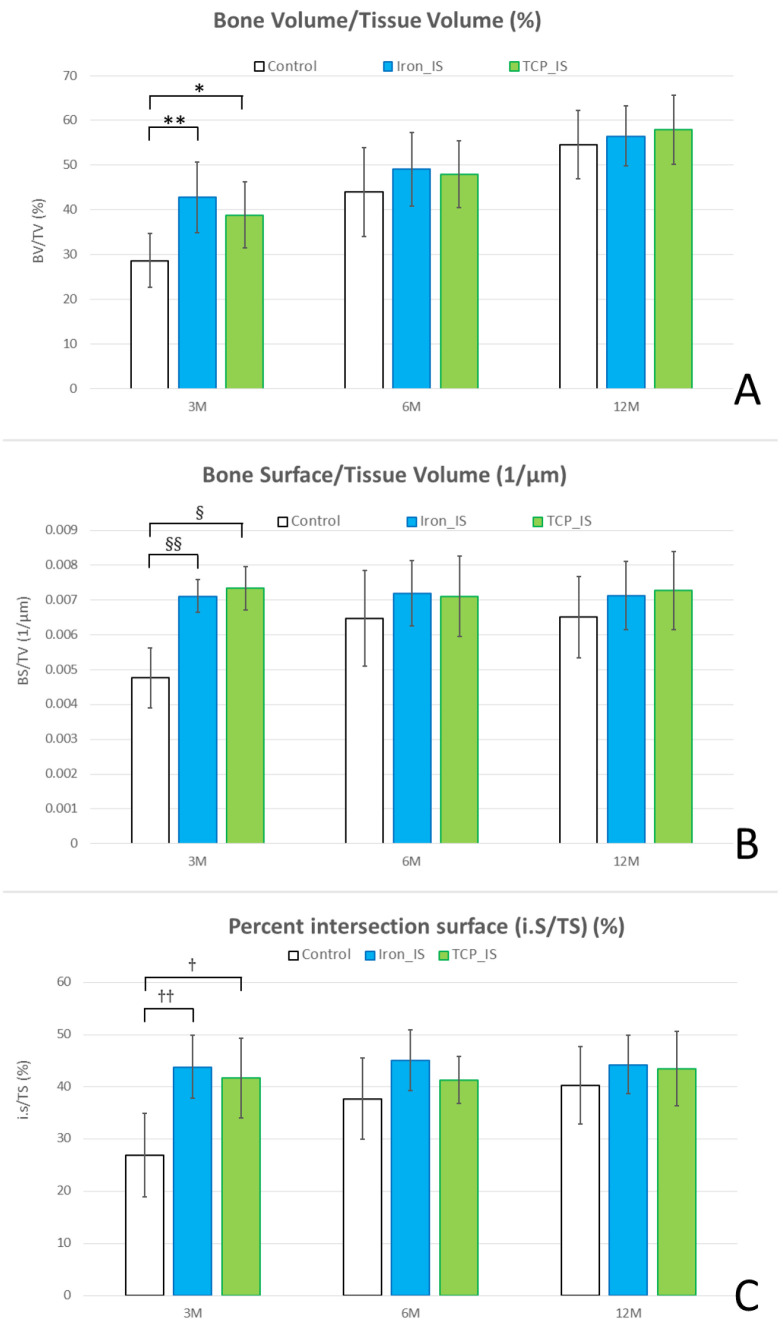
Quantitative evaluation of bone volume around the implants using micro-CT. Tissue volume (mm^3^), bone volume (mm^3^), percent bone volume (BV/TV; %), bone surface area (mm^2^), and bone surface area per total volume (BS/TV; 1/µm) were measured 0–1000 µm above the bone with the metallic implant, whereas intersection surface area (mm^2^), total surface area (mm^2^), and percent intersection surface (i.S/TS; %) were collected from 100 µm above the metallic implant. After 3 months of implantation, compared with the control group, the experimental groups (iron_IS and TCP_IS) demonstrated significantly higher (**A**) BV/TV (p = 0.0126 and 0.0432, respectively), (**B**) BS/TV (*p* = 0.0023 and 0.0009, respectively), and (**C**) i.S/TS (p = 0.0055 and 0.0174, respectively). Error bars represent standard deviations. *, **, §, §§, †, and †† denote significant differences between groups.

Micro-CT was performed to evaluate bone formation between the implants and bone tissues (Figure 4). Compared with the control group, the iron_IS and TCP_IS groups had higher BV/TV, BS/TV, and i.S/TS at post-implantation month 3, but not at post-implantation months 6 and 12. However, BV/TV, BS/TV, and i.S/TV did not differ significantly between the iron_IS and TCP_IS groups throughout the study period. Therefore, both iron_IS and TCP_IS resulted in favorable bone growth during the initial stage after implantation. The high BV/TV indicated a high degree of bone formation around the ROIs; in addition, the high BS/TV and i.S/TS indicated increased bone growth closer to the implant surface region. Our results demonstrated that compared with the control group, the experimental groups demonstrated better bone growth, particularly in the area adjacent to the implant surface.

**Figure 5:**
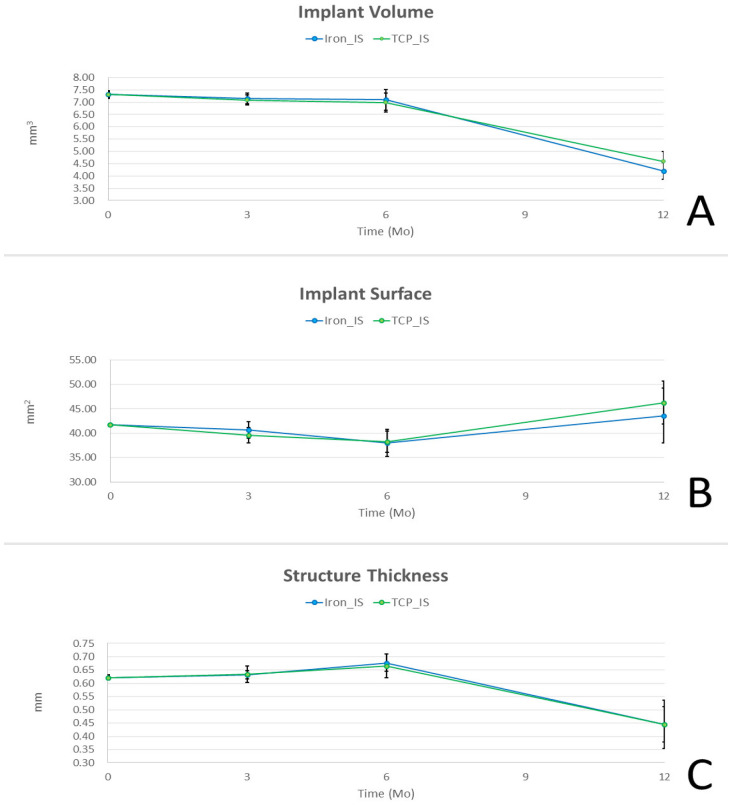
Degradation analysis of experimental interference screws using micro-CT based on their volume (IV; mm^3^), surface area (mm^2^), and structure thickness (Str_Thk, mm). The decrease in (**A**) IV and (**B**) implant surface area was slow during post-implantation months 0–6, but it accelerated later until post-implantation month 12. (**C**) Str_Thk slightly increased over post-implantation months 0–6 but decreased quickly over post-implantation months 6–12. Error bars represent standard deviations.

The results of our IS degradation analysis are presented in Figure 5. IV decreased gradually over post-implantation months 0–6, and it decreased further and more rapidly thereafter. In addition, implant surface area decreased slightly over post-implantation months 0–6 and then increased rapidly over the next 6 months. Str_Thk increased slightly over post-implantation months 0–6, but decreased rapidly over post-implantation months 6–12. These results suggested that after implantation, our Fe-based ISs gradually degraded into smaller fragments, reducing in Str_Thk and increasing TS.

### 2.4. Biochemical Analysis

**Figure 6:**
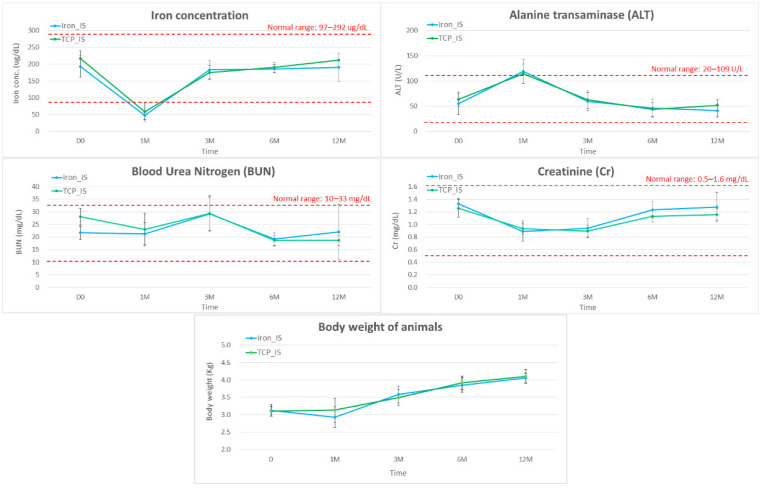
Changes in blood iron (µg/dL), alanine transaminase (ALT, U/L), blood urea nitrogen (BUN, mg/dL), and creatinine (Cr, mg/dL) concentrations and body weights of animals (kg) in months 0, 1, 3, 6, and 12 after implantation. Error bars represent standard deviations, and red dotted lines denote upper and lower limits.

For biochemical analysis, we collected blood samples from all the rabbits immediately pre-operatively and 1, 3, 6, and 12 months after implantation and determined blood iron, ALT, BUN, and Cr concentrations. The results are presented in Figure 6.

### 2.5. Histological and Histopathological Analyses

**Figure 7:**
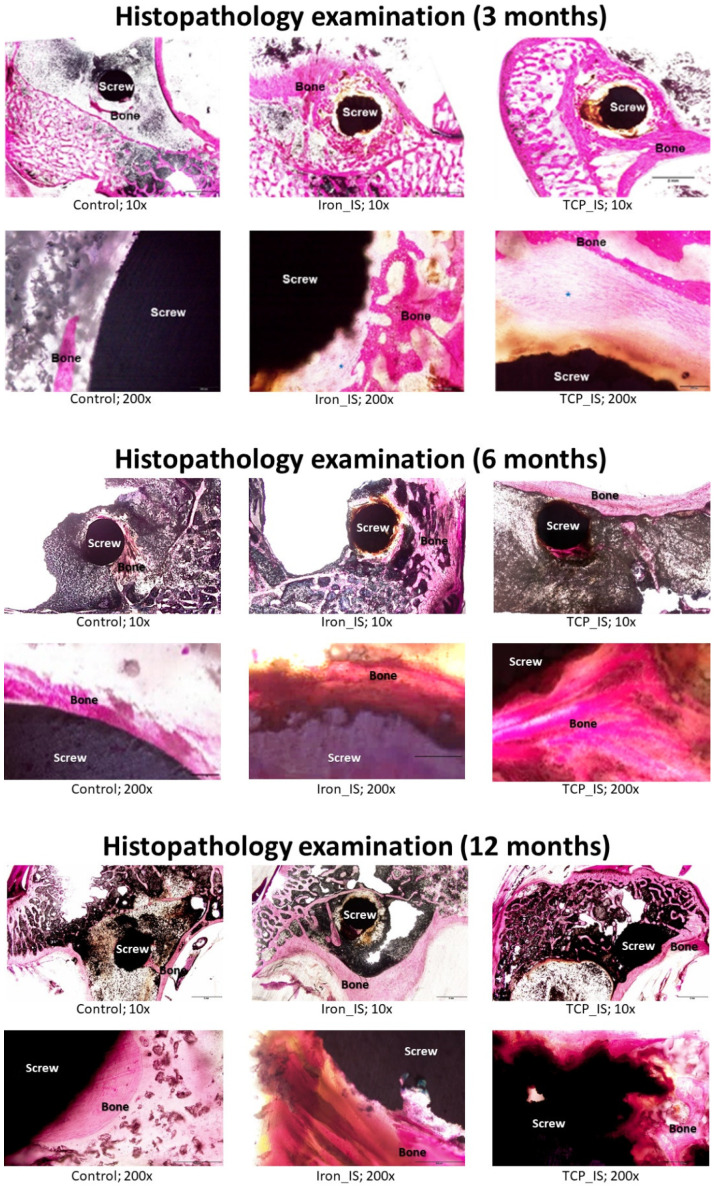
Histopathological examination of the bone–tendon–screw complex at months 3, 6, and 12 after implantation. Specimens were stained with Sanderson’s rapid bone stain and then counterstained with acid fuchsin.

Bone formation was observed at the interface of the host bone tissue and experimental ISs (Figure 7). Histological analysis, particularly at higher magnification, revealed new bone growth in the region in close contact with the experimental ISs. Our control group results corroborated those reported previously [27,28]. As such, our results revealed high biocompatibility of both iron_IS and TCP_IS, confirming the results of our previous study [26].

**Figure 8:**
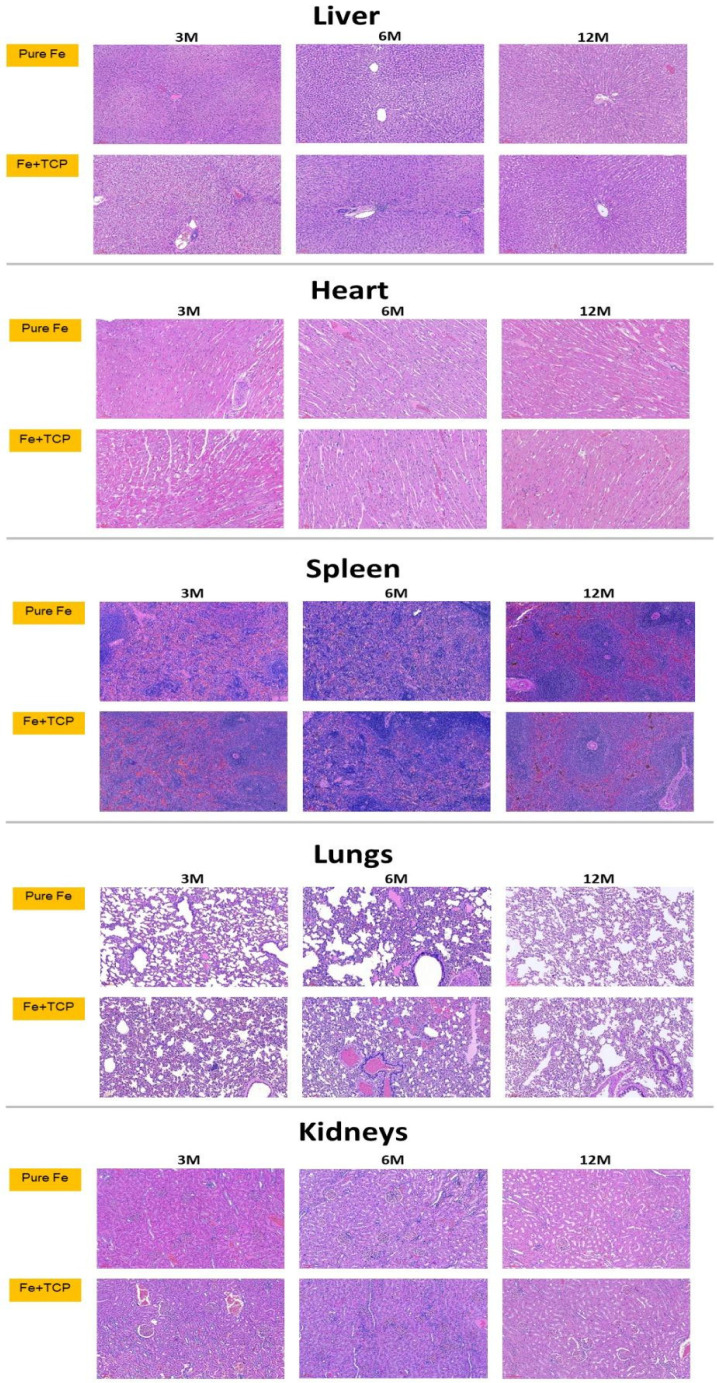
Histopathological specimens of visceral organs (liver, heart, spleen, lungs, and kidneys) obtained from the experimental groups at post-implantation months 3, 6, and 12. The liver, heart, lungs, and kidneys appeared to be normal, without visible pathological lesions during the entire study period. However, significant Fe deposition was noted in the spleen over time. Magnification, 100×.

**Figure 9:**
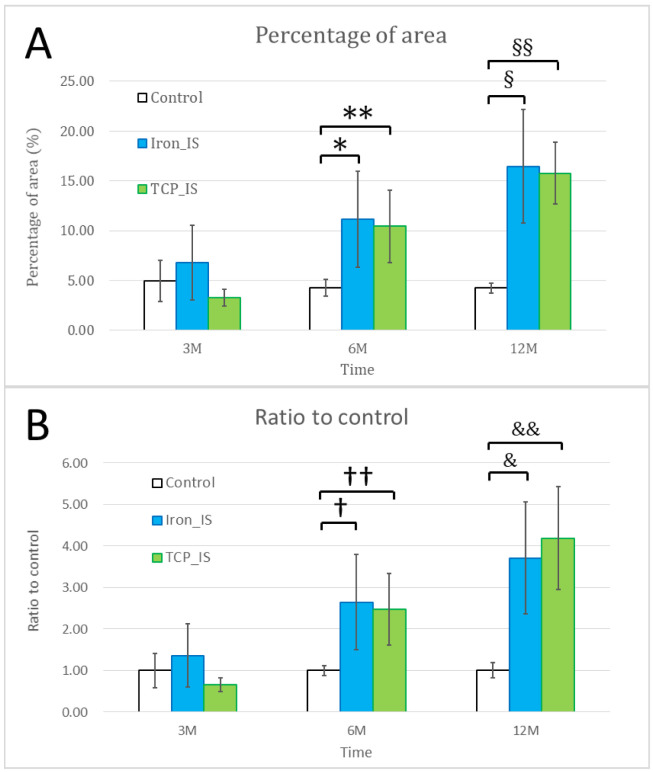
Semi-quantification of iron in spleen using Prussian blue special staining. (**A**) Percentage of area (%) was calculated as special stain area on a slide divided by the total specimen area on the same slide. (**B**) Ratio-to-control was calculated as percentage of area of experimental specimens divided by that of control specimens. Error bars represent standard deviations. *, **, §, §§, †, ††, &, and && denote significant differences between groups.

Figure 8 presents the histopathological findings of the liver, heart, spleen, lungs, and kidneys in both the experimental groups. The liver, heart, lungs, and kidneys appeared to be normal, without identifiable pathological lesions throughout the study period. Moreover, significant iron deposition was noted in the spleen in a time-dependent manner. Figure 9 presents the results of Prussian blue staining for semiquantitative estimation of iron deposition in the spleen.

## 3. Methods

### 3.1. IS Development Using AM

All IS specifications are presented in Figure 1A. Control ISs, procured from DePuy Synthes (Zuchwil, Switzerland), were standard stainless steel 2.7 mm cortex screws with a 12 mm screw-body length.

Two experimental ISs—with identical outline specifications and integrated porous structure—were produced using AM selective-laser sintering (SLM EOSINT M 270; EOS GambH-Electro Optical Systems, Krailling, Germany). The centerline average roughness (Ra) was 25 μm and the porosity rate was 15.4%. In particular, iron_IS was prepared using bioabsorbable spherical iron powder (purity > 99.5%) alone, whereas TCP_IS was prepared using 0.2 wt% TCP plus 99.8 wt% bioabsorbable spherical iron powder (purity > 99.5%) [29].

### 3.2. In Vitro IS Immersion Test

We performed an in vitro IS immersion test in Hank’s balanced salt solution (HBSS) to observe the changes in the gross appearance, iron concentration, pH level, and mass loss percentage over 12 months (Figure 1B–E and Table 1). Scanning electron microscopy (SEM) and X-ray photoelectron spectroscopy (XPS) analyses were also performed to evaluate the micro-appearance and element composition of the degraded ISs (Figure 2A,B, respectively).

### 3.3. In Vivo Animal Study Design

All animal experiments were approved by the Ethics Committee of the Biomedical Technology and Device Research Laboratories of Industrial Technology Research Institute and Institutional Animal Care and Use Committee of National Chiao Tung University in accordance with national animal welfare legislation (protocol numbers: ITRI-IACUC-2021-063M and NCTU-IACUC-109036), and the study protocol conformed to the National Institute of Health guidelines for the use of laboratory animals.

In total, 54 New Zealand white rabbits (mean body weight, 3.0 ± 0.5 kg; age, 6 months) were procured from the Master Laboratory (Taiwan). They were randomized to receive iron_IS, TCP_IS, or control IS by using a computer-generated randomization method (n = 18 per group). The ISs were implanted perpendicular to the mechanical axis of the tibia in the proximal tibia area of one of the stifle joints.

Each of the three groups was further divided into three subgroups (n = 6 per subgroup) based on the implantation periods (3, 6, or 12 months). At each time point, when histological analysis was performed in the six subgroup rabbits, the remaining 12 rabbits were analyzed using micro-CT and biomechanical testing. All animals received micro-CT immediately at the end of each experiment. All specimens were frozen for use in further biomechanical testing.

### 3.4. Surgical Methods

All surgical procedures were performed under general anesthesia administered using an intramuscular injection of 15 mg/kg zoletil (Virbac Taiwan, Taipei, Taiwan) + 0.05 mL/kg rompun (Bayer Taiwan, Taipei, Taiwan). To induce analgesia, the rabbits were given 0.15 mg/kg oral meloxicam (Metacam; Boehringer Ingelheim Taiwan, Taiwan) 1 day pre-operatively, immediately pre-operatively, and 2 days post-operatively.

Surgical procedures were modified from those reported by Yamakado et al. [30]. We have provided the details of the procedures elsewhere [20]. In brief, lateral parapatellar arthrotomy was performed in one of the stifle joints to easily expose the outer cortex of the lateral femoral condyle. The tendon of the extensor digitorum longus (EDL) muscle was identified and released from its insertion at lateral femoral condyle. A transverse tunnel (diameter, 2.0 mm) was then drilled perpendicular to the long axis of the tibia at the proximal tibia about 1 cm beneath the knee joint. The EDL tendon was then passed through the proximal tibial bone tunnel and transfixed with ISs during dorsiflexion of the paw. After correct placement of the screws, the anatomical layers (the joint capsule, muscles, subcutis, and cutis) were closed layer-by-layer with sutures (Vicryl 4–0; Ethicon, NJ, USA). The animals were returned to their cages after surgery and were allowed to move without any restriction or extremity immobilization. When they were sacrificed, all animals were found to be ambulant without signs of guarding or immobility; therefore, we assumed that the EDL tendon was functioning well. At the end of the experiment, all animals were euthanized with an intravenous overdose of pentobarbital. Their heart, liver, spleen, lungs, kidneys, and stifle joints were retrieved and stored at −20 °C until further study. For biochemical analysis, blood iron, alanine transaminase (ALT), blood urea nitrogen (BUN), and creatinine (Cr) concentrations were determined at post-implantation months 0, 1, 3, 6, and 12.

### 3.5. Biomechanical Analysis

Nine rabbits were sacrificed at post-implantation months 3, 6, and 12, and their stifle joints were used for biomechanical examination. The EDL tendon graft was harvested together with the proximal tibia. ElectroForce1 3510-AT (Bose Corporation—ElectroForce Systems Group, Eden Prairie, MN, USA) was used for biomechanical testing under the same conditions according to our previous study protocol [20]. The graft was secured at the load cell, while the tibia was fixed at the base plate. The parallel tensile load was gradually applied at a strain rate of 0.5 mm/min until gross failure on the myotendoninous junction. The ultimate failure loads of the constructs were recorded and analyzed.

### 3.6. Micro-CT Analysis

After the rabbits were sacrificed, six specimens were retrieved from each group 3, 6, and 12 months after implantation and scanned using a multi-scale nano-CT (Skyscan 2211; Bruker Micro-CT, Kontich, Belgium) at a voxel resolution of 18 μm. A 360° scan was then performed with a voltage of 160 kVp, a current of 80 μA, and an output of 6 W. Image reconstruction was performed using the reconstruction software program InstaRecon xCBR (version 2.0.4.6; InstaRecon, Urbana, IL, USA) and NRecon (Bruker Micro-CT). Ring artifact and beam-hardening correction were performed using NRecon (Bruker Micro-CT).

Reconstructed cross sections were reoriented, and the regions of interest (ROIs) were selected. We performed the analysis by using 1.4 mm images (113 slices). Thresholding and bone growth analysis were performed using CTAn. The threshold definition was based on implant so that the threshold of the same implant was the same; therefore, the images presented with the same pattern. The ROIs of the implant (diameter, 6 mm) were segmented; then, bone growth analysis was performed. The metallic structure and bone were separately isolated on the basis of the differences in X-ray absorption results. The border of the metallic structure was examined using CTAn with the shrink–wrap algorithm. Tissue volume (TV; mm^3^), bone volume (BV; mm^3^), percent BV (BV/TV; %), bone surface area (BS; mm^2^), and BS per TV (BS/TV; 1/mm) were measured 100–1000 µm above the implant bone. Moreover, the intersection surface area (i.S; mm^2^), total surface area (TS; mm^2^), and percent intersection surface (i.S/TS; %) were measured 100 µm above the implant.

Next, we performed implant degradation profile analysis. Here, a sphere-fitting measurement method was used to calculate the implant volume (IV; mm^3^), implant surface area (mm^2^), and implant structure thickness (Str_Thk; mm) [31,32,33]. Three-dimensional visualization was performed using Avizo (Thermo Fisher Scientific, Waltham, MA, USA) and CTVox (Bruker Micro-CT).

### 3.7. Histological Analysis

In each group, six specimens were retrieved for histological analysis. The proximal tibia, tendon graft, and implant were harvested together. The specimens were fixed in 10% formalin and decalcified in formic acid for 2 weeks. They were then dehydrated for at least 1 day and permeabilized for 5 days by using polymethylmethacrylate. After embedding, the samples were cut vertically, perpendicular to the long axis of the suture anchor, and at the level of the respective bone–implant interfaces. The sections were cut into slices approximately 150 μm in thickness by using a low-speed saw (IsoMet; Buehler, Lake Bluff, IL, USA) and ground to 60 μm by using a grinding and polishing machine. The ground sections were stained with Sanderson’s rapid bone stain (Dorn & Hart Microedge, Loxley, AL, USA) and then counterstained with acid fuchsin. Bone–tendon, tendon–implant, and bone–implant interfaces were examined with a light microscope.

Nine sets of visceral organs, namely the liver, heart, spleen, lungs, and kidneys, from each group were stained with hematoxylin and eosin (H&E), and the remaining sets were stained with Prussian blue special stain and semi-quantified using ImageJ (NIH, Bethesda, MD, USA). The percentage of area (%) was calculated as the special stain area on a slide divided by the total specimen area on the same slide. Moreover, the ratio-to-control was calculated as the percentage of area from the experimental specimen divided by that from control specimen.

### 3.8. Statistical Analysis

All experimental data are presented as means ± standard deviations, with values obtained from three experiments. A *p* value of <0.05 was considered to indicate statistical significance. The correlation was evaluated by determining Pearson and Spearman correlation coefficients. The Wilcoxon rank-sum test and Fisher’s exact test were used for nonparametric analysis. Data of more than two groups were compared using one-way analysis of variance and Tukey’s post hoc test for repeated measures. Statistical analysis was performed using SPSS (SPSS v.26, Chicago, IL, USA).

## 4. Discussion

We previously reported the short-term (3-month) biocompatibility and biological performance results of our AM manufactured Fe-based bioabsorbable implants in a living animal model [26]. In the current study, we not only extended the study period to 1 year, but also included one more experimental group (TCP_IS) to evaluate its biocompatibility and biological performance. As shown in Figure 1, the major corrosion of our Fe-based implants led to the formation of ferric oxide (Fe_2_O_3_)—corroborating the findings of previous studies [12,25]. Fe composites with 5 wt% TCP have been reported to have a slightly higher degradation rate than do pure Fe composites [23]. However, the degradation profiles of our implants were not affected by the addition of TCP (Figure 1C–E), possibly because TCP_IS contained extremely low concentrations of TCP. According to a previous report, although Fe-5 wt% TCP demonstrated better degradation rates, the cell viability tests, however, had unsatisfactory results [23]. We also failed at Fe-5 wt% TCP cell viability test by using AM technology, which is compatible with previous reports. After a repeated decrease in TCP concentration to 0.2 wt%, we were finally able to safely pass the cell viability test. In the future, finding the balance point of the TCP additive concentration between degradation profile and biocompatibility will be essential for bioabsorbable iron-based material.

In our in vivo biomechanical analysis, the ultimate failure loads were significantly higher than in the control group at post-implantation month 3, but not at post-implantation months 6 and 12 (Figure 3 and Table 2). At early stage after implantation, experimental screws demonstrated better bone growth than control screws, and was compatible with the previous report [26]. Moreover, no significant difference was noted between the iron_IS and TCP_IS group throughout the entire study period. This finding corroborated our previous results that AM-based ISs have better initial-stage biomechanical performance than conventional ISs [20]. In other words, as the bone–tendon–implant complex gradually healed in the long term, the biomechanical difference must have disappeared gradually. In addition, the addition of 0.2 wt% TCP did not appear to have improved the biomechanical performance of iron_IS. In summary, porous structure and surface roughness by AM production contributed better initial-stage biomechanical performance of experimental ISs [20,26].

Our micro-CT analysis results also corroborate those of our in vivo biomechanical analysis. Regarding bone formation, the iron_IS and TCP_IS groups demonstrated significantly higher BV/TV, BS/TV, and i.S/TV than did the control group at post-implantation months 3, but not at post-implantation months 6 and 12 (Figure 4). This result suggested that both iron_IS and TCP_IS facilitate bone formation, particularly in the areas close to the implants, resulting in improved biomechanical performance at the initial stage after implantation. However, no significant differences were noted in BV/TV, BS/TV, and i.S/TV between the two experimental groups. These findings might explain the results of our biomechanical analysis.

In our implant degradation analysis using micro-CT (Figure 5), IV and Str_Thk decreased significantly in the iron_IS and TCP_IS groups over post-implantation months 6–12 in both the experimental groups; however, Str_Thk increased slightly over the first 6 post-implantation months in both experimental groups. Moreover, in both the experimental groups, implant surface area increased over post-implantation months 6–12; however, the increase was significant only in the TCP_IS group. Nevertheless, the differences in IV, implant surface area, and Str_Thk between the iron_IS and TCP_IS groups were nonsignificant throughout the entire study period. These results indicated that IV and Str_Thk gradually decrease, but implant surface area gradually increases after the implantation of iron_IS or TCP_IS in the physiological environment; moreover, both iron_IS and TCP_IS demonstrated similar corrosion rates. Our initial implant centerline average roughness (Ra) was 25 μm and the porosity rate was 15.4%, as degradation progressed, roughness and porosity rate increased gradually. These findings were compatible with previous literature [3,12,26].

According to our in vivo biochemical analysis (Figure 6), most of the biochemical markers (iron, ALT, Cr, and BUN) were within normal range throughout the study period, except for the low iron and high ALT concentrations noted 1 month post-operatively. The low iron concentration was probably due to operative blood loss, and the high ALT concentration was possibly due to pain control and prophylactic antibiotic medications administered post-operatively. Both iron and ALT returned to their normal range at the next time point. According to the results of our histopathological analysis, all the retrieved visceral organs were normal, without detectable tissue toxicity. In the in vitro immersion test, iron_IS and TCP_IS demonstrated an average mass loss of 6.28 ± 4.95 and 6.06 ± 2.71 mg/month, respectively (Figure 1E and Table 1)—considerably lower than the physiological daily iron intake from diet [12,34]. This result, along with the result noted for blood iron concentrations, indicated that only few soluble iron ions were absorbed into the serum (Figure 6). However, semi-quantitative Prussian blue staining for iron demonstrated considerable iron accumulation in the spleen of the experimental groups at 6 and 12 months (Figure 9). All the aforementioned findings corroborate those of previous studies: clearance of iron-scaffold corrosion products might occur via the lymphatic system, and these products may enter the spleen via the circulatory system [34,35,36].

Our study has some limitations. First, the study duration was only 12 months. Although our in vivo biochemical and histopathological analyses demonstrated that the strong biocompatibility of our experimental ISs was sustained during all 12 months, future clinical studies examining the long-term (>1-year) biocompatibility of ISs are warranted. Second, the local and systemic degradation profiles, metabolic pathways, and maximal safety dose of iron also require exploration. In this study, we examined only the degradation profiles and possible iron metabolic pathways of our ISs using histopathological, semi-quantitative, and micro-CT analyses. Therefore, studies focusing on wider aspects of the iron degradation profile, particularly under different physiological conditions, are warranted. Third, we only used small animals, and their body weights gradually increased throughout the study period. However, the implant volume and weight were designed for human use. To obtain more accurate and realistic data, future studies should use animals with physiological parameters similar to those of humans.

In conclusion, the current results demonstrated that the bioabsorbable Fe-based ISs with pure iron or pure iron and TCP exhibit higher initial mechanical strength than do conventional metallic ISs. In addition, Fe-based ISs demonstrate high biocompatibility both locally and systemically after 12 months of implantation. However, corrosive iron products from the ISs may accumulate in the spleen. Nevertheless, given the mechanical superiority and high absorptivity afforded by our Fe-based ISs, Fe-based biodegradable materials may be strong candidates for application in the development of implantable medical device in the future.

## Figures and Tables

**Table 1:** In vitro immersion test results of iron_IS and TCP_IS implant weights.

	Day (s)	0	26	60	90	113	156	184	212	240	270	303	330	360
Implant weight (mg)	Iron_IS	420.00 ± 0.00	421.33 ± 7.64	419.67 ± 7.77	410.83 ± 8.86	405.13 ± 14.50	396.00 ± 19.95	390.63 ± 21.17	388.57 ± 15.71	380.07 ± 10.79	375.53 ± 11.67	368.80 ± 17.60	364.50 ± 23.95	344.67 ± 26.63
	TCP_IS	420.00 ± 0.00	420.90 ± 6.50	417.30 ± 5.70	410.20 ± 7.40	405.20 ± 12.90	394.90 ± 20.50	389.80 ± 23.00	384.70 ± 19.50	379.30 ± 21.90	372.10 ± 27.10	368.20 ± 26.10	356.20 ± 23.70	347.30 ± 28.10
Weight loss (mg)	Iron_IS	NA	−1.33	1.67	8.83	5.70	9.13	5.37	2.07	8.50	4.53	6.73	4.30	19.83
	TCP_IS	NA	−0.90	3.60	7.10	5.00	10.30	5.10	5.10	5.40	7.20	3.90	12.00	8.90
Average weight loss per month (mg/m)				IRON_IS		6.28 ± 4.95								
				TCP_IS		6.06 ± 2.71								

**Table 2:** Ultimate failure loads (N) of the bone–tendon–screw complex.

	Time (Months)		
Group	3	6	12
Control	25.90 ± 6.96	39.68 ± 6.63	47.53 ± 12.36
Iron_IS	37.61 ± 5.14	44.88 ± 6.70	51.76 ± 13.53
TCP_IS	39.51 ± 6.46	47.20 ± 8.60	54.62 ± 11.63
*p* value between groups			
Control vs. Iron_IS	0.0025	0.1560	0.5400
Iron_IS vs. TCP_IS	0.5374	0.5671	0.6702
Control vs. TCP_IS	0.0026	0.0919	0.2903

## Data Availability

The data sets generated and/or analyzed during the current study are not publicly available because they contain trade secrets, but can be made available from the corresponding author on reasonable request.
